# Active surveillance for *Theileria orientalis* and the invasive Asian longhorned tick (*Haemaphysalis longicornis*) in three Missouri beef herds

**DOI:** 10.1371/journal.pone.0319327

**Published:** 2025-04-01

**Authors:** Rosalie A. Ierardi, Savannah M. Chance, Celeste Morris, Jacqueline Nunnelley, Solomon O. Odemuyiwa, Angela B. Royal, Loren Schultz, Zhenyu Shen, Jordyn Young, Ram K. Raghavan

**Affiliations:** 1 Veterinary Medical Diagnostic Laboratory, University of Missouri, Columbia, Missouri, United States of America; 2 Department of Veterinary Pathobiology, College of Veterinary Medicine, University of Missouri, Columbia, Missouri, United States of America; 3 Division of Animal Sciences, College of Agriculture, Food and Natural Resources, University of Missouri, Columbia, Missouri, United States of America; 4 Department of Veterinary Medicine and Surgery, College of Veterinary Medicine, University of Missouri, Columbia, Missouri, United States of America; 5 College of Veterinary Medicine, University of Missouri, Columbia, Missouri, United States of America; 6 Department of Public Health, College of Health Sciences, University of Missouri, Columbia, Missouri, United States of America; University of Minnesota, UNITED STATES OF AMERICA

## Abstract

*Theileria orientalis* is a protozoan hemoparasite of cattle vectored by the rapidly emerging invasive Asian longhorned tick (*Haemaphysalis longicornis*). *Theileria*-associated bovine anemia (TABA) is easily mistaken for bovine anaplasmosis, which can lead to delayed diagnosis in areas where bovine anaplasmosis is endemic and TABA is newly emerging. Our objective was to surveil for infestation of cattle by *H. longicornis* and infection with *T. orientalis* on three Missouri cow-calf operations in counties where *H. longicornis* is known to be established. A total of 147 apparently healthy adult cows from 3 herds were inspected for ticks. Whole blood was collected for *T. orientalis* and *Anaplasma marginale* quantitative PCR and was also used for immediate preparation of blood smears and measurement of packed cell volumes. A total of 527 ticks were collected from the cows and taxonomically identified to the species level. Eighteen *H. longicornis*, including 9 adult females and 9 nymphs, were collected from 16 cows (Farm A, 2 cows; Farm B, 4 cows; Farm C, 10 cows). Intraerythrocytic *T. orientalis* organisms were presumptively identified on blood smears from 10 cows. Quantitative PCR screening of blood samples with primers designed to amplify all *T. orientalis* genotypes detected 11 positive samples (Farm A, 7 cows; Farm B, 3 cows; Farm C, 1 cow). Positive samples were re-tested with probes specific for the Ikeda, Chitose, and Buffeli genotypes, which detected the Chitose genotype in 10 samples and the Ikeda genotype in 1 sample. Detection of *T. orientalis* with concurrent infestation of cows by *H. longicornis* within these 3 herds, along with collection of *H. longicornis* from vegetation on the premises, supports local tick-borne transmission of this emerging pathogen.

## Introduction

The invasive Asian longhorned tick, *Haemaphysalis longicornis*, is an emerging concern to human and livestock health in the U.S. It is native to eastern Asia and Eurasia, and has been established as an invasive species in Australia and New Zealand for over a century [1]. Asian longhorned ticks (ALTs) were first recognized outside of quarantine in the U.S. on a New Jersey sheep in 2017 [2]. Subsequent review of archived tick specimens found individuals collected as early as 2010 [3]. ALTs have since been confirmed in 21 states and the District of Columbia [4], and ecological niche models predict further spread [5,6]. In Missouri, *H. longicornis* was first collected in June 2021 from vegetation in Greene County [7] and from a horse in Clay County [4]. Our research team has since identified first-time collections from vegetation in Linn County (August 2022), Boone County (April 2023), and Knox County (August 2023).

Invasive populations of *H. longicornis* are parthenogenetic, i.e., composed of asexually reproducing females, which greatly facilitates the tick’s ability to spread [1,8]. Additionally, *H. longicornis* feeds on a wide variety of hosts including humans, dogs, horses, cattle, white-tailed deer, and various other wildlife including several species of migratory birds [4,9]. Evidence suggests that *H. longicornis* can travel efficiently on domestic animals such as horses [8], dogs [8,10], and cattle [10], particularly in settings where animals are commingled such as shelters and livestock markets.

In addition to its importance as a livestock pest, *H. longicornis* is known to transmit pathogens associated with human disease in its native range [11], and laboratory studies indicate it is a competent vector for several human tick-borne pathogens found in North America including *Rickettsia rickettsii* (causative agent of Rocky Mountain spotted fever) [12], Powassan virus [13], and Heartland virus [14].

*Haemaphysalis longicornis* transmits a protozoal hemoparasite of cattle, *Theileria orientalis*, which causes a hemolytic anemia. Clinical signs of *Theileria*-associated bovine anemia (TABA, also known as oriental theileriosis) include inappetence, exercise intolerance, icterus, weight loss, and late-term abortions in pregnant cows [15]. TABA is easily mistaken for bovine anaplasmosis, a clinically similar disease caused by the rickettsial pathogen *Anaplasma marginale*, which can lead to delayed recognition of outbreaks in areas where bovine anaplasmosis is endemic and TABA is newly emerging [16]. Unlike bovine anaplasmosis, which can be treated with tetracyclines, TABA has no approved treatment. Burparvaquone has shown promise as an experimental treatment; however, long-lasting drug residues in meat and milk limit its usefulness in food-producing livestock [17]. In Australia and New Zealand, TABA has had a significant economic on the beef and dairy industries since its emergence in the 2010s [18–20], and likely has the potential to cost the U.S. cattle industry millions of dollars annually.

*Theileria orientalis* has 11 distinct genotypes, the most clinically relevant of which are Ikeda, Chitose, and Buffeli. Ikeda is the most virulent and can cause severe disease in up to 6% of infected cattle [21], with reported case fatality rates up to 16.7% [22]. Notably, *T. orientalis* Ikeda occasionally causes severe disease in calves < 6 months old [23], which is not a characteristic of bovine anaplasmosis.

*Theileria orientalis* Ikeda genotype was first detected in the U.S. in a Virginia beef herd in 2017 [16], and has since been confirmed in at least 14 states [24]. Sporadic detections of *T. orientalis* in Missouri cattle have been reported since June 2023, but there is limited data on the extent of its spread. Our objective was to surveil for infestation of cattle by *H. longicornis* and for infection with *T. orientalis* on three Missouri cow-calf grazing operations in counties where we have previously found *H. longicornis* to be established.

## Materials and methods

### Study herds

**Fig 1:**
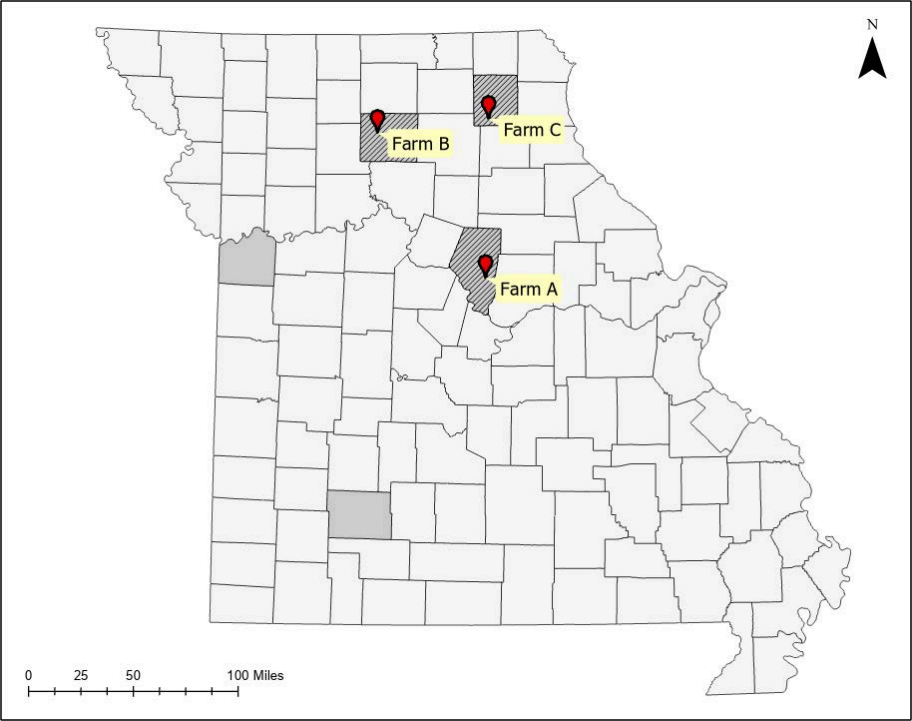
County map of Missouri with approximate locations of the study farms. Shaded counties indicate where ALTs have been reported, but are not known to be established. Hatched counties indicate where ALTs are known to be established. Map created in ArcGIS Pro 2.4.0 with public domain TIGER/Line Shapefiles (tl_2010_29_county10; tl_2020_us_zcta510) downloaded from the U.S. Census Bureau.

University of Missouri (MU) Agricultural Experiment Station facilitated access to three University-owned herds in central and north-central Missouri. All 3 herds raise their own replacements and did not report purchasing new cattle within the last 3 to 4 years. Farm A offers a feed-out program for steers which are shipped in from around Missouri; these steers are housed in confinement (open-sided barns with access to dry lot) and are not allowed to access pastures or otherwise have direct contact with the rest of the Farm A herd. Farm locations, herd sizes (number of adult cows and replacement heifers), and dates sampled are indicated in Table 1 and Figure 1. Sample collection was performed under the supervision of licensed veterinarians (Ierardi, Morris) with the approval of the University of Missouri Animal Care and Use Committee (Protocol 44642).

**Table 1:** Locations, herd and sample sizes, and sampling timeframe of study farms.

	County	Herd size (*N*)	Sample size (*n*)	Date sampled
Farm A	Boone	220	52	April 23–24, 2024
Farm B	Linn	350	54	May 31, 2024
Farm C	Knox	75	41	June 3, 2024

### Sample size calculation

We calculated sample size in a manner designed to detect presence, rather than estimate prevalence, based on the assumption that *T. orientalis* is still relatively rare in the midwestern U.S. We chose a sample size that would allow detection of *T. orientalis* with 95% confidence at a prevalence of ≥ 5%. Sample size is calculated as shown [25],


$$n=\left(1-{\left(\alpha \right)}^{\frac{1}{D}}\right)\left(N-\frac{D-1}{2}\right)$$ (1)

where *α* is 0.05 (1 – 95% confidence level), *N* is the number of animals in each herd, and *D* is the minimum expected number of affected animals (*N* ×  minimum expected prevalence).

### Sample collection and processing

Cows were inspected for ticks by palpating (“scratching”) commonly infested areas of the body for a maximum of 5 minutes with the cows restrained in a manual or hydraulic chute with a headgate. Ticks were removed from cattle with forceps and placed in vials with 1–2 mL of absolute ethanol for preservation and transport to the laboratory. Vials were labeled with identification codes that allowed ticks to be matched with the individual cow, farm, and date of collection. Ticks were subsequently removed from the ethanol, identified under dissecting microscopes with standard taxonomic keys [26,27], separated by species, sex, and life stage, and thereafter stored at -80°C. Ticks identified as *H. longicornis* were kept in ethanol and shipped to the Ecology and Entomology Team, Rickettsial Zoonoses Branch, Division of Vector-Borne Diseases, National Center for Emerging and Zoonotic Infectious Diseases, Centers for Disease Control and Prevention (Atlanta, GA) for official confirmation of species identification.

From each cow, blood was collected from the median caudal (tail) vein with a 1.5-inch 18- to 20-gauge needle attached to a vacutainer hub. EDTA-anticoagulated tubes and additive-free tubes of 3 to 5 mL blood each were transported to the laboratory on ice. Packed cell volumes (PCVs) were measured with EDTA-anticoagulated whole blood within 12 hours of collection. Whole blood was subsequently frozen at −80°C until quantitative PCR (qPCR) analysis was performed. Blood in additive-free tubes was allowed to clot and was refrigerated overnight for centrifugation and analysis the next morning.

### *Theileria orientalis* qPCR

DNA was extracted from 200 µL of whole blood using the GeneJET Genomic DNA Purification Kit (Thermo Fisher Scientific, Waltham, MA, USA) according to the manufacturer’s instructions, with a final elution volume of 200 µL. qPCR was performed in duplicate using primers and probes as previously described [28,29], summarized in Table 2.

**Table 2:** Primers and probes used for *Theileria orientalis* qPCR.

Oligonucleotide	Sequence (5′–3′)
*T. orientalis* forward primer	5′-GCAAACAAGGATTTGCACGC-3′
*T. orientalis* reverse primer	5′-TGTGAGACTCAATGCGCCTAGA-3′
Universal probe	5′-FAM-TCGACAAGTTCTCACCAC-MGB-NFQ-3′
Ikeda probe	5′-Cy5-CATGGACAGTGCTTGGC-MGB-NFQ-3′
Chitose probe^*^	5′-SUN-TCCTCRGCGCTGTTCT-MGB-NFQ-3′
Buffeli probe	5′-FAM-CTCCTTTGCAGTATTCTTCTATCTC- MGB-NFQ-3′

* The “R” placeholder indicates an approximately equimolar mixture of A and G, included to account for a polymorphism in the Chitose sequence [29].

First, a pair of universal primers and a universal probe were used to amplify the major piroplasm surface protein gene (*MPSP*) of any and all *T. orientalis* genotypes that might be present. Primers and the universal probe were diluted to a working concentration of 5 µM. For the universal qPCR, amplification was completed with an Applied Biosystems StepOnePlus (Thermo Fisher Scientific, Waltham, MA, USA) using standard cycling and the following run method: 95°C for 10 min followed by 45 cycles consisting of 15 sec at 95°C and 60 sec at 60°C. Ct baseline threshold was set at 0.02 for analysis of all runs.

Second, positive samples were re-tested in duplicate with the same primers and specific probes to distinguish between the Ikeda, Chitose, and Buffeli genotypes. Primers and genotype-specific probes were diluted to a working concentration of 10 µM. For the genotype-specific qPCR, amplification was completed with an Applied Biosystems 7500 (Thermo Fisher Scientific, Waltham, MA, USA) using standard cycling and the following run method: 95°C for 10 min followed by 45 cycles consisting of 15 sec at 95°C and 60 sec at 60°C. Ct baseline threshold was set at 0.02 for analysis of all runs.

For universal and genotype-specific qPCR, each singleplex reaction consisted of 10 µL of master mix (QuantiTect Probe qPCR Kit; Qiagen, Hilden, Germany), 1 µL each of forward and reverse primer, 1 µL of probe (Integrated DNA Technologies, Coralville, IA, USA), 4.5 µL of molecular biology grade water, and 2.5 µL of DNA template in a 20 µL total reaction volume. A sample was defined as “positive” when both reactions yielded a Ct value of < 35 and both amplification curves were appropriately sigmoid-shaped. Synthetic DNA sequences were used as positive controls (gBlocks Gene Fragments; Integrated DNA Technologies, Coralville, IA, USA). The “universal” *T. orientalis* positive control sequence was modified from published sequences of the *MPSP* gene and has 92.5–100% identity to the first 100 *T. orientalis* isolates of all 3 genotypes listed in GenBank. Genotype-specific positive control sequences were modified from previously published [29] genotype-specific regions of the *MPSP* gene (GenBank accessions: Ikeda, KM624620; Chitose, KM624619; Buffeli, KM624621). Modifications included addition of base pairs to the 3′ and 5′ ends to internalize primer binding sites and/or sequence modifications to avoid the formation of secondary hairpin structures. Molecular biology grade water was used as a negative control. Positive and negative controls were included in duplicate on all runs. Positive control sequences are listed in S2 Table.

### Blood smear review

Blood smears were prepared immediately from whole blood by a single investigator (Nunnelley) in the field, according to standard methods [30]. Prepared slides were air-dried, transported to the laboratory, and stained using an automated Siemens Hematek 3000 slide stainer (Siemens Healthineers, Erlangen, Germany) with Wright-Giemsa within 12 hours. A clinical pathology resident (Nunnelley) and a board-certified clinical pathologist (Royal) independently reviewed all slides for the presence of hemoparasites. Both investigators were blinded to molecular and serologic test results for each subject. A minimum of ten 100 × objective fields were assessed for the presence of hemoparasites; many slides required review of more numerous 100 × objective fields for confident assessment, particularly in slides with any degree of stain precipitate. Semiquantification of hemoparasites was reported as an approximate number of organisms per 100 × objective field. After independent blood smear reviews were completed, any discrepancies were jointly reviewed by both investigators and consolidated into agreed-upon finalized results.

### *Anaplasma marginale* qPCR

Genomic DNA extracted for *T. orientalis* qPCR was also used for *A. marginale* qPCR. Samples were tested in duplicate using primers and a probe designed to amplify the *msp1b* gene and which are sensitive and specific for detection of *A. marginale* [31]. Each reaction consisted of 12.5 µL of master mix (QuantiTect Probe PCR Kit; Qiagen, Hilden, Germany), 1 µL each of *A. marginale* forward and reverse primer and 1 µL of *A. marginale* probe (Integrated DNA Technologies, Coralville, IA, USA), 1 µL of 25 mM magnesium chloride (Thermo Fisher Scientific, Waltham, MA, USA), 3.5 µL of molecular biology grade water, and 5 µ L of DNA template in a 25 µL total reaction volume. Amplification was completed with an Applied Biosystems StepOnePlus (Thermo Fisher Scientific, Waltham, MA, USA) using standard cycling and the following run method: 95°C for 15 min followed by 45 cycles consisting of 15 sec at 95°C and 60 sec at 60°C. A sample was defined as “positive” when both reactions yielded a Ct value of < 35 and both amplification curves were appropriately sigmoid-shaped. Ct baseline threshold was set at 0.02 for analysis of all runs.

### *Anaplasma marginale* cELISA

Serum samples were submitted for *A. marginale* cELISA at the University of Missouri Veterinary Medical Diagnostic Laboratory (VMDL). Samples were tested with a commercially available kit (*Anaplasma* Antibody Test Kit, cELISA v2; VMRD, Pullman, WA, USA) per manufacturer’s instructions. A positive result was defined as ≥ 30% inhibition.

### Analysis of field-collected ticks

We did not analyze ticks collected from cattle for the presence of *T. orientalis*, as there is a significant risk of spurious detection within the host-derived blood meal, even in ticks that may not be competent vectors [32]. However, we did have ALTs which were collected from pastures on the study farms in 2023–2024 and stored at −80°C. Specimens were collected from vegetation via dragging 750-meter transects in accordance with CDC’s published guidelines [33].

Prior to DNA extraction, *H. longicornis* ticks were separated by life stage, site, and collection season into pools of 1 adult female or up to 5 nymphs. Surface decontamination of tick pools was achieved by vortexing for 1 min in 1 mL of 3% bleach, followed by vortexing for 1 min in 1 mL of sterile distilled water [34,35]. Tick pools were homogenized in a Fisherbrand Bead Mill 24 (Thermo Fisher Scientific, Waltham, MA, USA) for 90 seconds at 4 m/s with six 3.175 mm stainless steel beads (Lysing Matrix S; MP Biomedicals, Irvine, CA, USA) suspended in 0.5 mL of Dulbecco’s phosphate buffered saline. DNA was extracted from 100 µL of tick pool homogenate using the GeneJET Genomic DNA Purification Kit (Thermo Fisher Scientific, Waltham, MA, USA) according to the manufacturer’s instructions for mammalian tissue, with the addition of overnight incubation at 56°C in digestion buffer/proteinase K for the initial digestion step [36]. The final elution volume was 200 µL. Tick pool DNA was stored at −20°C until subjected to universal *T. orientalis* qPCR as previously described.

## Results

A complete list of individual cow-level results is provided in S1 Table.

### Tick collection and identification

A total of 527 ticks were collected from the cattle (Table 3), with a mean of 2.6 ticks/cow on Farm A, 2.3 ticks/cow on Farm B, and 6.5 ticks/cow on Farm C. Most ticks were found between the udder and thighs, behind the elbows, and below the tailhead. Occasional specimens were collected around the ears, chin, and brisket; however, many of the cows did not tolerate handling around the head and it is therefore likely that some ticks in these locations were missed. Eighteen *H. longicornis* – 9 adult females and 9 nymphs – were collected from 16 cows (Farm A, 2 cows; Farm B, 4 cows; Farm C, 10 cows). In transit from Farm B, the labels fell off 3 vials containing a total of 3 ticks (all *Amblyomma americanum* adults) and therefore these 3 ticks could not be traced back to individual cows.

**Table 3:** Species identification of ticks collected from cattle on the 3 study farms.

	Farm A	Farm B	Farm C	Total
*Amblyomma americanum* (lone star tick)	131	110	65	306
*Dermacentor variabilis* (American dog tick)	0	10	191	201
*Haemaphysalis longicornis* (Asian longhorned tick)	2	4	12	18
Total	134^*^	125^**^	268	527

* 1 tick from Farm A was an adult *Ixodes* but was too damaged to identify at the species level.

** 1 tick from Farm B was too damaged to be identified

### *Theileria orientalis* qPCR

Eleven cattle were positive for *T. orientalis* (Table 4), corresponding to detection rates of 2.4% on Farm C, 5.6% on Farm B, and 13.5% on Farm A. Ten of 11 cows were infected with the Chitose genotype, with only 1 cow positive for Ikeda. The Buffeli genotype was not detected in any of the cows.

**Table 4:** Summary of results for confirmed *T. orientalis*-positive samples.

Sample	*T. orientalis* qPCR Ct values				*T. orientalis* blood smear	Am qPCR	Am cELISA
	Universal	Chitose	Ikeda	Buffeli			
Farm A 07	24.6	26.5	ND	ND	(+)	(+)	(+)
Farm A 24	27.1	29.1	ND	ND	(+)	(+)	(+)
Farm A 25	27.6	30.7	ND	ND	(−)	(+)	(−)
Farm A 30	25.4	27.8	ND	ND	(+)	(+)	(+)
Farm A 31	25.5	28.2	ND	ND	(+)	(+)	(−)
Farm A 44	23.7	26.5	ND	ND	(+)	(−)	(−)
Farm A 45	22.8	25.3	ND	ND	(+)	(+)	(+)
Farm B 09	28.7	30.8	ND	ND	(−)	(+)	(+)
Farm B 51	25.9	28.2	ND	ND	(−)	(+)	(+)
Farm B 53	25.5	27.7	ND	ND	(+)	(+)	(+)
Farm C 10	24.9	NA	30.1	ND	(+)	(−)	(+)

*All Ct values are reported as the mean of duplicate samples. Am =  *Anaplasma marginale*. ND =  no detection.

### Blood smear review

**Fig 2:**
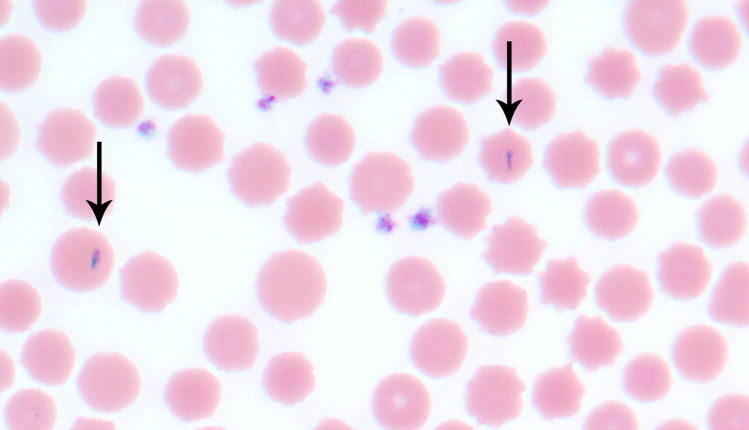
*Theileria orientalis* organisms within bovine erythrocytes (arrows). Rare (typically < 1 per 100 × objective field) erythrocytes contain slender and elongated protozoa, 2 to 4 µm in length, with basophilic cytoplasm and a pink-purple internal region.

*Theileria* organisms were presumptively detected on 10 of 146 blood smears (Figure 2; Table 4). The smear for Farm B 30 was inadvertently lost. Eight of the 10 positive smears were from cattle with a positive *T. orientalis* qPCR result. Two of the 10 positive smears were from cattle with a negative *T. orientalis* qPCR result (Farm B 1; Farm B 2); both of these 2 samples were positive for *A. marginale* via qPCR and cELISA. Compared to *T. orientalis* qPCR, blood smear review in our study yielded a sensitivity and specificity of 72.7% and 98.5%, respectively. *Anaplasma marginale* was not detected on any smear.

### *Anaplasma marginale* qPCR and cELISA

*Anaplasma marginale* was detected by qPCR in 67 of 147 samples (45.6%), and anti-*Anaplasma* antibodies were detected by cELISA in 74 of 147 samples (50.3%). Of the *T. orientalis*-positive samples, 9 were also positive for *A. marginale* by qPCR and 8 were seropositive for *Anaplasma* spp. by cELISA (Table 4).

### Packed cell volumes

Packed cell volumes of all cows except one were within normal limits, between 26–41% (Farm A, mean =  32.2%; Farm B, mean =  33.5%; Farm C, mean =  35.8%). Cow 15 on Farm B was anemic with a PCV of 18% and was found dead several days after sample collection. *Theileria orientalis* was not detected by blood smear or qPCR. *Anaplasma marginale* was detected by qPCR (Ct 22.9) but no organisms were identified on blood smear. No necropsy was performed.

### Analysis of field-collected ticks

We analyzed 13 pools of 26 *H. longicornis* ticks collected from pastures on the study farms May 2023–August 2024 (Table 5). One adult female was lost due to cracking of the microcentrifuge tube during bead homogenization and could not be analyzed. An additional 22 nymphs were unavailable for analysis since initial collections in each county were shipped to the CDC for official confirmation and were not returned. No amplification was detected in any *H. longicornis* tick pools with universal *T. orientalis* qPCR.

**Table 5:** Description of *H. longicornis* ticks collected from vegetation on study farms 2023-2024 and available for *T. orientalis* qPCR.

Pool	No. of ticks	Life stage	Site	Time collected
HL3	4	Nymphs	Farm A	May–July 2023
HL4	1	Adult female	Farm A	July 2023
HL5	1	Adult female	Farm A	July 2023
HL6	1	Adult female	Farm A	July 2023
HL7	1	Adult female	Farm A	July 2023
HL8	1	Adult female	Farm A	July 2023
HL13	3	Nymphs	Farm A	April 2024
HL14	3	Nymphs	Farm A	April–May 2024
HL1	3	Nymphs	Farm B	May–July 2023
HL2	1	Adult female	Farm B	July 2023
HL9	3	Nymphs	Farm B	May 2024
HL10	3	Nymphs	Farm C	June 2024
HL11 *	1	Adult female	Farm C	August 2024
HL12	1	Adult female	Farm C	August 2024

*Tube cracked during bead homogenization and the sample was lost. No qPCR performed.

## Discussion

We detected *T. orientalis*, an emerging agent of bovine infectious anemia, in three Missouri cow-calf herds. Detection of *T. orientalis* Ikeda was our primary concern, as this genotype is the most clinically relevant; however, it was important to distinguish between the Chitose and Buffeli genotypes as well. Although the Buffeli genotype appears to be rare and does not usually cause clinical signs, it has been sporadically detected in U.S. cattle herds since 1950, including a Missouri beef herd in 1999 [37].

In Virginia, surveillance at livestock markets recently detected the Ikeda and Chitose genotypes in 8.7% and 1.8%, respectively, of 1,980 cattle tested [38]. Multiple studies in Australia have also found Ikeda to be the dominant genotype [39,40]. Notably, in our sample, Chitose was the most frequently detected genotype. Prospective re-testing of these herds would be valuable to see how the proportion of detected genotypes changes over time.

Previously, our research team identified *H. longicornis* on the three study farms in August 2022 (Farm B), April 2023 (Farm A), and August 2023 (Farm C). Ticks were found while dragging pastures during the spring and summer months as a component of a pre-existing research project in 2022–2024. On all three sites, identification of at least six individual ticks and/or multiple life stages within a single year meets USDA’s criteria for local establishment [4,41]. Although we have consistently detected *H. longicornis* on these premises, they account for < 1% of total ticks collected by dragging. We have not yet encountered the abundance of *H. longicornis* ticks described by researchers doing field surveillance in other states [42,43].

*Theileria orientalis* was not detected in any of the *H. longicornis* ticks collected from pastures on the study farms May 2023–August 2024. However, the available sample size of 26 specimens is too small to detect *T. orientalis* at an assumed prevalence of 5%, even with 80% confidence, and therefore analysis of a larger number of ticks would be necessary to draw meaningful conclusions about the degree to which local *H. longicornis* populations may be infected with *T. orientalis*. Notably, researchers in Virginia have detected *T. orientalis* in approximately 13% of host-seeking *H. longicornis* nymphs [42].

*Haemaphysalis longicornis* accounted for 3.4% of the ticks we collected from the study cattle. Cattle on the study farms were not heavily tick-infested, with 26 being the largest number of ticks collected from a single cow (Farm C 31, mostly *Dermacentor variabilis*). All farms reported deworming cattle within the prior 6 months with products containing moxidectin or doramectin, both of which can substantially reduce tick numbers of cattle [44,45]. The farms also mow (“brush hog”) pastures periodically to control weeds, which may also reduce local tick populations [46].

In our study, the sensitivity of blood smear review for detection of *T. orientalis* in comparison to qPCR was 72.7%, significantly higher than a previously published estimate of 38.7% [39]. This likely reflects meticulous review of slides by two independent observers, and an investment of time that would be impractical for routine use. Our intention was not to use blood smears as a screening test, but rather to allow assessment of parasite morphology. The 2 false positives (i.e., presumptive identification of *Theileria* organisms on a smear with a negative qPCR result) likely represent misidentification of stain precipitate, which is easily mistaken for the linear forms of *T. orientalis*. Our findings indicate that, while impractical for use as a herd-level screening test, blood smears can be highly specific when interpreted by trained observers and are a valuable tool in the diagnostic workup of individual sick cattle.

While all cows were apparently asymptomatic at the time of sample collection, one cow (Farm B 15) had a PCV of 18% and was found dead several days after sample collection. *Theileria orientalis* was not detected, and acute anaplasmosis seems unlikely given the presence of a robust antibody titer (42% inhibition) and an *A. marginale* Ct value not significantly different from clinically unaffected cows in the same herd. Farm B has had several cases of bacillary hemoglobinuria in the last few years, a disease characterized by hemolytic anemia and sudden death, so this may be a worthwhile differential. Unfortunately, no necropsy was performed.

While it is unknown how *T. orientalis* Chitose and Ikeda entered these 3 herds, a few hypotheses can be considered. Cattle are frequently shipped to the Midwest from all over the U.S., including the Appalachian region where *T. orientalis* Ikeda is currently becoming endemic [47,48]. Modeling studies indicate the spread of a novel infectious disease is highly likely, particularly when infections are not recognized quickly, carrier animals remain in the population, and movements are unrestricted, all of which apply to the epidemiology of *T. orientalis* Ikeda [49]. Farm A offers an annual program where steers from other farms can be fed to slaughter weight, which could create a potential avenue for *T. orientalis*-infected cattle and/or *H. longicornis* ticks to enter the premises. While these steers are kept in confinement and strictly separated from the rest of the herd, rabbits and nesting birds are often observed in the barns, which could potentially facilitate movement of ticks from housed cattle to the rest of the farm.

Alternatively, ticks could be brought onto the farms by a number of wildlife species. White-tailed deer are locally abundant on and near each of the three farms, and are well-documented hosts for *H. longicornis*; however, their role in the direct transmission of *T. orientalis* appears to be minimal [50].

In areas with extensive cattle movements and well-established *H. longicornis* populations, such as Australia and New Zealand, *T. orientalis* Ikeda can quickly reach a within-herd prevalence in excess of 75% once introduced [51,52]. The epidemiology of *T. orientalis* Ikeda in the U.S. is less well characterized and is expected to vary regionally depending on abundance of *H. longicornis* and cattle movements. Given the presence of at least 1 *T. orientalis* Ikeda-infected cow on Farm C, we hope to re-test these cattle to see how the prevalence changes over time.

The relatively low detection rates of *T. orientalis* suggest these 3 herds were sampled early in the course of local spread. It may also suggest a low level of tick-borne transmission, which would be consistent with the low numbers of *H. longicornis* we have collected, although high within-herd prevalences have been reported in areas where tick activity was apparently minimal [53]. It remains uncertain whether other local tick species are capable of transmitting *T. orientalis*. The lone star tick, *Amblyomma americanum*, is abundant in Missouri and is a competent vector of *Theileria cervi* in deer [54]; additional work is urgently needed to determine whether this species is capable of vectoring *T. orientalis*.

Finally, mechanical transfer of blood-contaminated fomites, such as reusing needles during herd vaccinations, is a well-documented mode of transmission for *A. marginale* [55] and appears to be a likely mode of transmission for *T. orientalis* as well [56]. Iatrogenic transmission should be considered as another possible route of exposure in these 3 herds.

In summary, we identified *T. orientalis*-infected cows and *H. longicornis* ticks feeding on cows in the same herd, along with collection of *H. longicornis* from pastures on the premises. Our findings indicate that local tick-borne transmission of bovine theileriosis is likely in these 3 herds.

## Supporting Information

S1 Table Complete table of detailed results at the individual cow level. The table includes duplicate qPCR Ct values, cELISA percent inhibition, number of ticks collected by species and life stage, percent packed cell volumes, and *T. orientalis* blood smear results. Three rows (Unknown1, Unknown2, Unknown3) include tick counts corresponding to the 3 unlabeled vials from Farm B.
(XLSX)

S2 Table Nucleotide sequences of the gBlocks used as positive controls for *T. orientalis* qPCR. (XLSX)
